# Enteral Delivery of Pravastatin Sodium Tablets: Effect of Compounding into a Liquid Form and Co-Administration of Enteral Nutrition

**DOI:** 10.3390/pharmacy12010032

**Published:** 2024-02-09

**Authors:** Serena Logrippo, Roberta Ganzetti, Matteo Sestili, Diego Romano Perinelli, Marco Cespi, Giulia Bonacucina

**Affiliations:** 1 Hospital Pharmacy, Santa Maria della Stella Hospital, USL Umbria 2, 05018 Orvieto, Italy; serena.logrippo@sanita.marche.it; 2 Hospital Pharmacy, Engles Profili Hospital, AST Ancona, 60044 Fabriano, Italy; 3 Hospital Pharmacy, Carlo Urbani Hospital, AST Ancona, 60035 Jesi, Italy; roberta.ganzetti@sanita.marche.it; 4 Territorial Pharmaceutical Service, AST Ancona, 60035 Jesi, Italy; matteo.sestili@sanita.marche.it; 5 CHIP Building, School of Pharmacy, University of Camerino, 62032 Camerino, Italy; diego.perinelli@unicam.it (D.R.P.); giulia.bonacucina@unicam.it (G.B.)

**Keywords:** pravastatin sodium, enteral formulas, tablet compounding, fibers

## Abstract

Background: Compounding solid oral dosage forms into liquid preparations is a common practice for administering drug therapy to patients with swallowing difficulties. This is particularly relevant for those on enteral nutrition, where factors such as the administration procedure and co-administration of enteral nutrition play crucial roles in effective drug delivery. Due to the limited studies focused on this practice, the impact of co-administered nutrition remains unclear. Methods: Pravastatin tablets were compounded into two liquid formulations and administered through three independent tubes for ten cycles. The drug amount was quantified upstream and downstream of the tubes both with and without different (fiber content) nutritional boluses. Results: The compounding procedure did not lower the drug amount with respect to the original tablets. However, when the liquid formulation was pumped through the tubes, a statistically significant reduction in the pravastatin administered (between 4.6% and 11.3%) was observed. The co-administration of different nutritional boluses or the compounding procedure did not affect the general results. Conclusions: Pravastatin loss appears unavoidable when administered via the enteral tube. Although, in this case, the loss was of limited clinical relevance, it is important not to underestimate this phenomenon, especially with drugs having a narrow therapeutic index.

## 1. Introduction

The pharmaceutical market offers numerous formulations to address various patient needs. While the majority of commercially available drugs are formulated as solid oral dosage forms (e.g., tablets, capsules, powders, or granules for oral use) [1], they may not be suitable for certain patient groups. Specifically, pediatric, geriatric, bedridden, non-compliant patients, or individuals with swallowing complications may benefit from alternative dosage forms other than solids [2,3]. Currently, a common practice in hospital or home care settings involves modifying solid oral dosage forms by crushing tablets or opening capsules. The resulting powder or content is then dispersed in a suitable vehicle [4,5,6], such as water or food beverages, allowing patients to take their drug therapy in a liquid formulation.

A critical consideration relates to patients who are entirely incapable of swallowing, often due to the natural aging process or various primary pathologies (e.g., post stroke, central nervous system disorders, temporary disabilities). In such cases, these patients rely on enteral tubes (e.g., naso-gastric tubes, NGTs) as the sole route for both feeding and drug therapy administration [7]. Consequently, meals and medications must be prepared in a suitable form to facilitate administration through an enteral tube.

The effectiveness of drug administration through enteral tubes has been the subject of numerous investigations, particularly concerning the use of proton pump inhibitors [8]. Studies by Sostek et al. [9] and Freston et al. [10] compared the pharmacokinetic profiles of esomeprazole and lansoprazole, respectively, finding comparable results. However, Kotake et al. [11] observed a significant reduction in the blood concentration of the drug amiodarone when administered via an enteral tube. While comparing pharmacokinetic data is practical, it fails to pinpoint the specific sources of differences. The process of administering therapy through enteral tubes involves multiple steps, including drug manipulation, absorption in the polyurethane of the tube, interactions with the meal within the tube, and/or substances in the gastrointestinal tract. Additionally, patients requiring nasogastric intubation may exhibit variations in drug absorption due to specific pathological conditions [11]. Presently, there are only a few in vitro studies focusing on medicine administration via an enteral tube. Clark-Schmidt et al. [12] studied the effect of the flushing solution composition and volume as well as the tube size on carbamazepine suspension administration. They found a significant loss of the drug, mainly as a function of the initial concentration of the drug suspension. Cacek et al. reported similar results for phenytoin administration through nasogastric tubes [13]. Ruzsíková et al. evaluated various dosage forms containing different active pharmaceutical ingredients (APIs) and diverse administration procedures. In all the cases studied, some drug loss was observed during enteral tube administration, with the type of dosage forms and administration procedures playing significant roles [14]. Furthermore, enteral tubes serve not only for therapy administration but also for patient nutrition. Residues from nutrition may adsorb part of the liquid formulations used for therapy administration, altering the effective amount of drugs administered. While some drug–nutrition interactions are reported (for example, warfarin with vitamin K or other nutrition components [15,16], or levodopa with a high-protein diet [17]), distinguishing between interactions within the tube (drug absorption on food residues) and specific interactions affecting drug absorption in the gastro-enteric mucosa remains challenging [18].

The aim of this work is to thoroughly investigate the administration of a compounded medicinal product through feeding tubes using an in vitro model. This approach should enable the evaluation of the effectiveness of drug administration (the effective amount of drug administered), also pinpointing whether any concerns arise from the compounding process or the administration procedure. Pravastatin sodium was selected as the model drug because it is a widely prescribed medication for the elderly population (enteral tube feeding being more prevalent among the elderly [19,20]), and is exclusively available in Italy in tablet form [21]. Furthermore, our interest extended to understanding the potential influences that nutritional formulas could exert on the amount of drug reaching the gastro-enteric tract, as an interaction leading to dose reduction cannot be ruled out a priori [9]. Once again, Pravastatin sodium represents a good model drug, given that statins are typically administered after the evening meal and, generally, following tube flushing with water. Considering the market’s diverse array of nutritional formulas, catering to a wide range of patient needs [22], we opted for two commonly used formulas with a balanced intake of nutrients and similar compositions, differing only in their fiber content. Fibers exhibit a high absorption capacity for water and drugs [23,24], potentially representing a significant factor in determining the actual amount of pravastatin administered.

## 2. Materials and Methods

### 2.1. Materials

Immediate-release tablets containing 20 mg of pravastatin sodium (PraNa) (Pensa Pharma s.p.a., Milano, Italy) were bought in local pharmacies, while PraNa raw material was kindly donated by Teva Pharmaceutical Industries Ltd., (Debrecen, Hungary) through Angelini A.C.R.A.F. s.p.a. (Roma, Italy). Methyl and propyl para hydroxy benzoate sodium (Galeno s.r.l., Carmignano, Italy) and sodium bicarbonate (Sigma Aldrich, Steinheim, Germany) were all pharmaceutical grade.

Nutricomp^®^ Standard Fiber enteral nutrition was purchased from B. Braun Melsungen AG (Melsungen, Germany), while Nutrison^®^ 1 Kcal/mol nutrition was from Nutricia Advanced Medical Nutrition (Danone Group, Milano, Italy). In this manuscript, the Nutricomp^®^ Standard Fiber (fiber content 1.5% *w*/*v*) will be reported as NSF (nutrition standard fiber), while the Nutrison^®^ 1 Kcal/mol (fiber content 0% *w*/*v*) will be reported as NWF (nutrition without fiber). Details of the composition of the two nutrition formulas are provided in Table S1 in the Supplementary Materials.

Polyurethane feeding tubes (length 120 cm, inner and outer diameters 5.4 mm and 4 mm, respectively, catheter size 12 French) were purchased from Teleflex Medical s.r.l. (Varedo, Italy). Polypropylene syringes for enteral use were bought from Pentaferte Italia s.r.l. (Campli, Italy).

Distilled water was prepared using a Sartorius AG System (Goettingen, DE, Germany). Throughout this manuscript, the term water always refers to distilled water.

### 2.2. Sample Preparation

Two liquid preparations containing PraNa at a concentration of 2 mg/mL were prepared from commercial tablets. The first formulation, referred to as the extemporaneous preparation (EXT), was created by crushing a single PraNa tablet (20 mg PraNa) in a mortar and subsequently adding 10 mL of water to achieve a homogeneous liquid preparation. This method represents the common procedure employed in hospital wards [25,26]. The second formulation, named the galenic preparation (GAL), was prepared following the method previously outlined by Bonacucina et al. [26]. In brief, 10 tablets of PraNa were dispersed under stirring in 80 mL of a buffered preserved aqueous solution [26,27] in a volumetric flask. The final volume was adjusted to 100 mL by adding the same medium. The buffered preserved aqueous solution contains sodium bicarbonate at 8.4% and methyl and propyl para-hydroxybenzoate at concentrations of 1.5 and 0.5 mg/mL, respectively.

In addition to EXT and GAL, a further liquid preparation, named standard solution (STD), was prepared by applying the same procedure as for the GAL preparation, but PraNa tablets were substituted with pure PraNa powder.

### 2.3. In Vitro Model Study

The in vitro model designed to study the enteral administration of compounded preparations as liquid formulations via a feeding tube represents a modified version of that used by Ruzsíková et al. [8]. It was structured as follows: three independent tubes were positioned and secured on a support at a 45° angle to mimic the physiological insertion angle of the tube in a patient. This arrangement was chosen to simulate three dysphagic patients receiving enteral nutrition. Ten consecutive administrations of the drug liquid preparation were delivered into each tube. Following each drug administration (10 mL of a liquid formulation with a drug concentration of 2 mg/mL), the tube was flushed with 10 mL of water. Due to the flushing, the final theoretical downstream concentration became 1 mg/mL, which is half of the theoretical concentration upstream.

The same setup was replicated to examine the impact of different nutritional formulas on enteral drug administration. Given that statin administration typically occurs after the evening meal, the sequence of operations differed: a 300 mL nutritional bolus corresponding to a single meal was introduced into the tube using a syringe, and the tube was subsequently rinsed with 10 mL of water [28]. Subsequently, 10 mL of PraNa liquid preparation (with an upstream drug concentration of 2 mg/mL) were administered into the tube and collected in a burette. Finally, the tube was rinsed with 10 mL of water, and the liquid was collected into the same burette. Since only the liquid drug formulation and its flushing water were collected, the final theoretical PraNa concentration was 1 mg/mL. This procedure was repeated ten times for each of the three tubes.

### 2.4. Spectrophotometric Determinations of PraNa Concentration

The quantitative analysis of the drug was conducted using UV–Vis spectrophotometry (Shimadzu UV-1800, software UV-Probe 2.43) at the specific wavelength for PraNa, which is 238 nm. The absorbance of the samples was measured, and the concentrations were determined utilizing a calibration curve. The calibration curve, constructed with PraNa solutions at various concentrations (22.5–5 μg/mL), exhibited a coefficient of determination (R^2^) of 0.999. For the GAL and STD preparations, the UV–Vis analysis presented challenges due to parabens exhibiting an absorbance peak at 256 nm, interfering with the PraNa quantification. Consequently, a dual-wavelength spectrophotometric method was employed in the presence of parabens, measuring the absorbance exclusively related to PraNa. The method’s validity was confirmed using solutions of known concentrations of PraNa and parabens in water.

Quantification was performed both before (upstream samples) and after (downstream samples) administration, following appropriate dilutions: 1:100 (for the upstream concentration) and 1:50 (for the downstream concentration), respectively. These dilutions account for the different upstream and downstream concentrations, given that the theoretical upstream concentration was 2 mg/mL, while the final downstream concentration was 1 mg/mL due to tube flushing with 10 mL of water.

Furthermore, the drug content of the commercial tablets was determined in randomly collected samples from the same batch and compared with the amounts declared by the manufacturer.

### 2.5. Statistics

The data for a single formulation (EXT, GAL, or STD) for an individual feeding tube are presented as the mean and standard deviation. The data for a single formulation across all three feeding tubes are aggregated and reported as the pooled mean (*Mp*), utilizing the weighted arithmetic mean, also known as the grand mean, and the pooled standard deviation (*SDp*). The *SDp* has been calculated using the method known as “the exact pooled variance” [29]:

(1) $$SDp=\sqrt{\frac{\sum_{i=1}^{k}{{(n}_{i}\cdot SD}_{i}^{2})+\sum_{i=1}^{k}{n_{i}\cdot (M}_{i}-{Mp}_{i})2}{\sum_{i=1}^{K}n_{i}}}$$

where *n_i_*, *M_i,_* and *SD_i_* are the sample size, mean, and standard deviation, respectively, of the i-th subset.

The comparisons between the different samples were carried out using a one-way ANOVA with a significance level of *p* < 0.05. If statistically significant differences were observed, post hoc Tukey or Dunnett tests were applied (a family-wise level of significance of 5% was chosen). Prior to the ANOVA, the normality and homoscedasticity were evaluated with the Shapiro–Wilk normality test and Brown–Forsythe test, respectively. 

The absence of any growing or decreasing trend in the successive administrations of the compounded preparations was evaluated by performing a linear regression for each dataset (downstream amount of PraNa vs. administration sequence) and testing the significance of the calculated slope (significance level of *p* < 0.05). 

All the statistic tests as well as the regression analysis and evaluation were performed with the software Prism v 6.01 (GraphPad Inc., Boston, MA, USA).

## 3. Results and Discussion

### 3.1. Effect of the Compounding on PraNa Administration

The impact of the compounded procedure on the PraNa administration was assessed through the spectrophotometric determination of PraNa concentrations both upstream and downstream of the feeding tube. 

**Figure 1:**
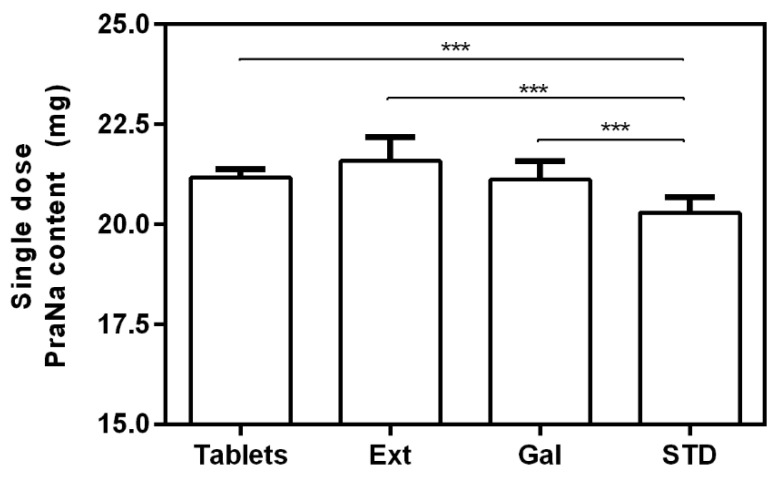
Mean values and standard deviation (*n* = 10) of the single-dose PraNa content on the commercial tablets and on the three formulation prepared. The asterisks represent the significance of the Tukey’s test according to the following: *** *p* < 0.001.

Initially, the accuracy of the compounded procedure was evaluated by comparing the PraNa content in tablets with that in their compounded formulations, namely EXT and GAL. The average PraNa content in the tablets was 21.17 ± 0.22 mg, exhibiting an average variation of 5.9% with respect to the nominal drug content of 20 mg. The PraNa tablets demonstrated compliance with the European Pharmacopeia in terms of the uniformity of mass and content for single-dose preparations [30]. The average PraNa content for a single dose (10 mL) of compounded formulations was 21.59 ± 0.59 mg for EXT and 21.12 ± 0.59 mg for GAL, respectively. A one-way ANOVA, followed by a Tukey test, was employed to verify the differences in the PraNa content between the commercial tablets and the single dose of their compounded formulations. The statistical analysis also included the PraNa content of the standard formulation (average content of PraNa 20.27 ± 0.38 mg), prepared from the PraNa powder rather than the commercial tablets. No statistically significant differences were found between the two compounded formulations and the commercial tablets, while the STD formulation exhibited a PraNa content statistically lower than that of all the other samples (Figure 1). This suggests that the selected compounding procedures do not result in any loss of PraNa compared to the tablet content. In this case, compounding appears to ensure the accuracy of the drug amount, aligning with previously published results [26]. The differences with the standard formulation are exclusively attributed to the PraNa content in this preparation (20.27 ± 0.38 mg), which is much closer to the nominal value (20 mg) compared to that of the commercial tablets (21.17 ± 0.22 mg).

**Figure 2:**
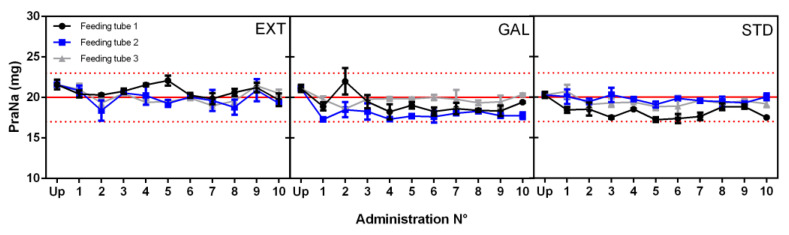
PraNa amount recovered after each single administration for the three formulations and for all the feeding tubes. On the *x* axis, Up represents the upstream PraNa amount for each formulation. The error bars refer to precision (expressed as SD) of the absorbance measurement (for each single sample the absorbance has been measured in triplicate). The solid red lines represent the nominal PraNa amount of a single dose (20 mg), while the upper and lower dotted red lines define the range 85–115% with respect to 20 mg.

The three prepared formulations, namely EXT, GAL, and STD, were subsequently administered through the feeding tubes, and after flushing, downstream concentrations of PraNa were determined. This process was sequentially repeated ten times. The results of each individual administration for each tube and formulation are reported in Figure 2. It is evident that no single administration resulted in a downstream concentration outside the range of 85–115%, which represents the European Pharmacopeia (EP) limits of drug content for commercial tablets (depicted by the dotted red line in Figure 2, corresponding to a range of 17–23 mg). It is crucial to note that the EP limits of the uniformity of the content of the single-dose preparations assay refer to commercial tablets and not to compounded formulations. However, since commercial tablets serve as the starting point for the compounding procedure, compliance with such an assay provides a valuable indication of the quality of the entire process.

**Figure 3:**
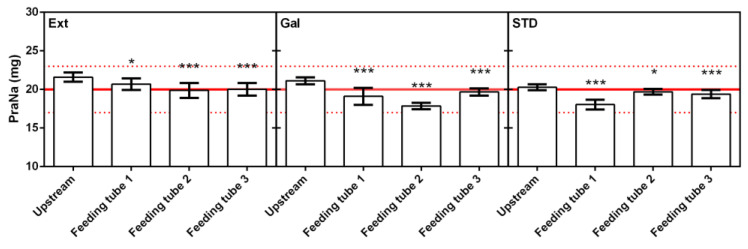
Mean and standard deviation (*n* = 10) values of PraNa administered (upstream) and recovered (downstream) for each tube and formulation. The solid red line represents the nominal PraNa amount of a single dose (20 mg), while the upper and lower dotted red lines define the range 85–115% with respect to 20 mg. The asterisks represent the significance of the Dunnet’s test (upstream is the reference group) according to the following: * 0.05 < *p* < 0.01; *** *p* < 0.001.

Beyond assessing the quantity of the PraNa administered, it is crucial to examine the presence of specific trends in the data. The existence of a particular trend could signify that the drug remains trapped in the tube (or is absorbed in the polyurethane), resulting in a reduction in the administered PraNa amount as successive administrations take place. Analyzing the data in Figure 2, no trends were observed in the repeated administrations within the same tube. This was confirmed by performing a linear regression for each dataset and testing the significance of the calculated slopes. Consequently, the observed variations during the repeated administrations can be attributed solely to chance. In this context, it becomes possible to assess the differences between the amount of drug administered (upstream) and the amount recovered at the end of each tube (downstream). The comparison between the mean values recorded upstream and downstream is reported in Figure 3. Despite the mean amount of PraNa administered never falling outside the European Pharmacopeia (EP) limits of drug content (85–115%, marked by the dotted red line in Figure 3), there is consistently a statistically significant reduction (confirmed by Dunnet’s test) in the amount of drug recovered downstream compared to its content in the compounded preparation. Hence, it appears that the administration of PraNa, both as compounded formulations (EXT and GAL) and a liquid solution (STD) through an enteral feeding tube, consistently results in a certain loss of the drug, which is within the range of 4.6% to 10.6%. This aligns with a qualitative and quantitative equivalent result (around 10% for uncoated tablets) obtained by Ruzsíková et al. [14], who used a similar in vitro model (although relying on gravimetric quantification rather than spectrophotometric determination) and a compounding procedure corresponding to EXT. 

### 3.2. Nutritional Formulas’ Influence on PraNa Administration

**Figure 4:**
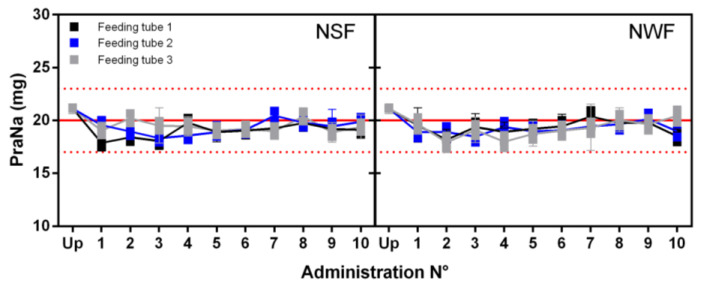
PraNa amount recovered after each single administration for the GAL formulation preceded by the flushing of alimentary bolus for all the feeding tubes. On the *x* axis, Up represents the upstream PraNa amount in the GAL formulation. The error bars refer to precision (expressed as SD) of the absorbance measurement (for each sample, the absorbance has been measured in triplicate). The solid red lines represent the nominal PraNa amount of a single dose (20 mg), while the upper and lower dotted red lines define the range 85–115% with respect to 20 mg. Each single experimental point is represented by a box where the lower limit is the mean of the PraNa content calculated from the adjusted absorbance and the upper one is the PraNa mean derived from the uncorrected absorbance.

**Figure 5:**
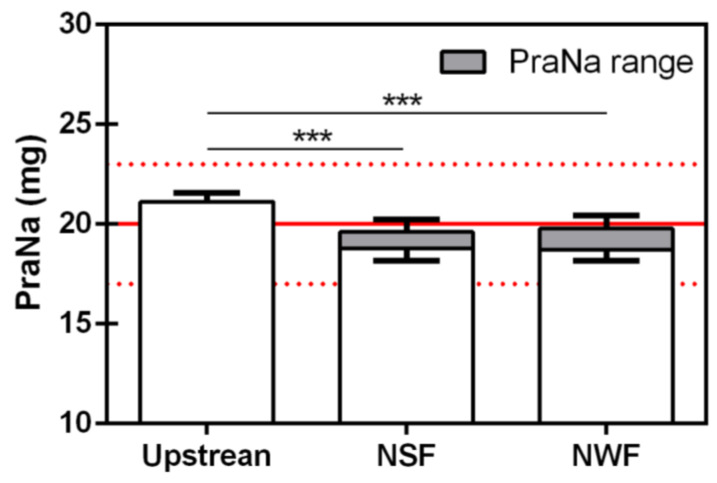
PraNa amount recovered after each single administration for the GAL formulation preceded by the flushing of alimentary bolus for all the feeding tubes. On the *x* axis, Up represents the upstream PraNa amount in the GAL formulation. The error bars refer to precision (expressed as SD) of the absorbance measurement (for each sample, the absorbance has been measured in triplicate). The solid red lines represent the nominal PraNa amount of a single dose (20 mg), while the upper and lower dotted red lines define the range 85–115% with respect to 20 mg. Each single experimental point is represented by a box where the lower limit is the mean of the PraNa content calculated from the adjusted absorbance and the upper one is the PraNa mean derived from the uncorrected absorbance. The asterisks represent the significance of the Tukey’s test according to the following: *** *p* < 0.001.

**Figure 6:**
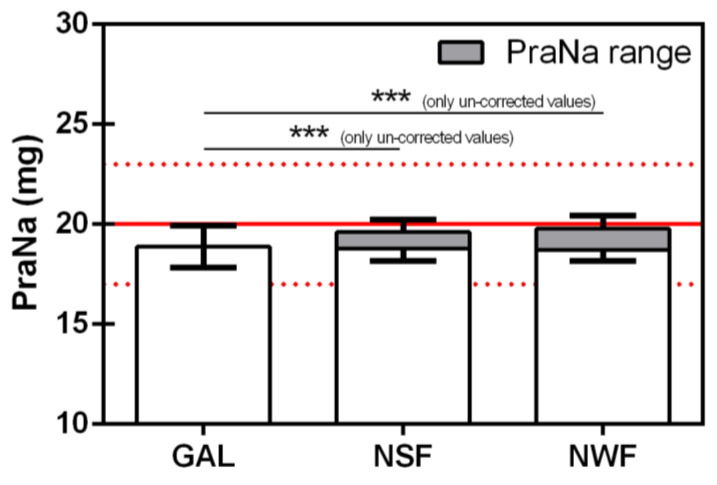
Pooled mean and pooled standard deviation values of PraNa recovered (downstream) for the galenic formulation with the two different nutritional formulas (NSF and NWF) and without (GAL). The solid red line represents the nominal PraNa amount of a single dose (20 mg), while the upper and lower dotted red lines define the range 85–115% with respect to 20 mg. The asterisks represent the significance of the Tukey’s test according to the following: *** *p* < 0.001.

The results obtained from the previous experimental model regarding PraNa administration are valuable for a preliminary screening of different formulations or to evaluate any significant loss of drug due to tube absorption or accumulation. However, this model overlooks a potentially relevant variable—the presence of residues of other substances previously administered through the same enteral tube. An enteral tube is not solely used for therapy administration but also for patient nutrition. The residues of nutrition, characterized by a complex composition and variable state of hydration, could adsorb part of the liquid formulations used for therapy administration, causing a reduction or oscillation in the effective amount of the drug reaching the stomach. To evaluate the effect of food residues, a study was conducted where each PraNa administration was preceded by a standard amount of a meal (300 mL). Two commercial nutrition formulas, one containing a standard amount of fibers (NSF) and the other without them (NWF), were included in the study due to the high absorption power of fibers, both toward water and drugs [23,24]. As all the compounded formulations yielded almost equivalent results (Section 3.1), the study on the effect of food intake was exclusively conducted using the GAL formulation. The measurements of the drug downstream concentrations were performed after flushing the alimentary bolus (300 mL), washing the tube with 10 mL of water, administering the PraNa single dose (GAL), and following this with an additional 10 mL of water to mimic a realistic care setting. Nutrition residues could introduce a source of error during the absorbance measurements of the PraNa solution. A preliminary study conducted without the PraNa formulation showed that meal administration followed by 10 mL of water resulted in the recording of a certain absorbance at 258 nm during UV analysis. The recorded absorbance corresponds to around 6% and 4% of the PraNa content in the single doses, for NWF and NSF, respectively. Therefore, when quantifying PraNa, it is necessary to consider a potential overestimation of a maximum of 6% and 4%. For this reason, in the subsequent graphs (Figure 4, Figure 5 and Figure 6), the amount of PraNa recovered will be reported as a box, representing the range of possible PraNa content. In the box, the lower limit is the mean of the PraNa content calculated from the adjusted absorbance (considering the maximum absorbance increases due to the nutrition residues), and the upper limit is the PraNa mean derived from the uncorrected absorbance. It is very likely that for each measurement, the actual PraNa content lies between these two limits.

The PraNa content of each single administration for each feeding tube is depicted in Figure 4. Regardless of the type of nutrition used, the amount of recovered PraNa consistently falls within the range of 85–115% relative to the nominal dose. Moreover, after linear regression, the *p*-values for the slopes were consistently higher than 0.05, indicating the absence of any trend in the data. Consequently, the recovered amount of PraNa in each individual instance appears to be independent of the administration sequence.

The final pooled mean (Mp) of the PraNa amount, considering both the corrected and uncorrected absorbance values, was 18.78 ± 0.60 mg and 19.60 ± 0.62 mg for NSF, and 18.72 ± 0.56 mg and 19.78 ± 0.66 mg for NWF. These results, depicted in Figure 5, were comparable to those obtained considering only the compounded formulations: there is consistently a statistically significant reduction in the amount of PraNa administered (both considering the corrected and uncorrected values) compared to the upstream values. In all cases, the nutrition formulas do not appear to significantly affect the amount of drug recovered. 

The comparison between the downstream amounts of PraNa recovered after the administration of the GAL with and without the preliminary administration of the nutritional bolus (the analysis has been performed considering both the corrected and the uncorrected values) is presented in Figure 6. The ANOVA results showed a statistically significant increase in the PraNa amount only when the drug was administered after the nutritional bolus, and the absorbance values were not corrected. Thus, the preliminary administration of a nutritional bolus never reduces the amount of compounded PraNa administered through an enteral tube, at least for the procedure reported here.

## 4. Conclusions

This study demonstrated that for the PraNa immediate-release tablets, the product obtained with the standard compounding procedure (EXT) as well as with the galenic one (GAL) produced equivalent results when administered via an enteral feeding tube. The co-administration of the enteral nutrition did not affect the concentration of the active substances reaching the gastrointestinal tract, and these results remained consistent regardless of the administration sequence.

However, a certain amount of drug is consistently lost during the administration process. Considering all the different compounding procedures and experimental conditions (with or without nutrition), the amount of drug lost, from the compounding process up to the enteral feeding tube administration, was approximately 9.5%, ranging from 4.6% to 11.3%. This drug loss appears unavoidable and is exclusively related to the administration process via the enteral tube, which is the most critical step.

Although, in the case of the PraNa administration in this study, the drug loss was of limited relevance, it is important not to underestimate this phenomenon as a general rule. Considering that the effective content of the active molecule in commercial solid oral dosage forms could range between 115 and 85% of the nominal dose, the amount of drug effectively administered, in the worst-case scenario, could be lower than 20–25% of its nominal content. This scenario appears worrisome, especially in the case of medicines with a narrow therapeutic index. In such cases, specific studies should be conducted to assess their compounding and administration through enteral tubes, precisely quantifying the drug loss. Furthermore, future studies aimed at identifying appropriate strategies to mitigate drug loss would be important.

## Data Availability

Data are contained within the article and Supplementary Materials.

## Supplementary Materials

The following supporting information can be downloaded at https://www.mdpi.com/article/10.3390/pharmacy12010032/s1, Table S1: Composition of the two nutrition formulae used in the study as provided by the manufacturers.
