# Supplementary material for: Enteral Delivery of Pravastatin Sodium Tablets: Effect of Compounding into a Liquid Form and Co-Administration of Enteral Nutrition
Source: Pharmacy (Basel). 2024 Feb 9;12(1):32. doi: 10.3390/pharmacy12010032 (PMC10892702; doi:10.3390/pharmacy12010032)
Supplement: Supplementary file 1 [file pharmacy-12-00032-s001.zip › pharmacy-2845813-supplementary.pdf]

Supplementary Material

of

Enteral Delivery of Pravastatin Sodium Tablets: Effect of Compounding into a Liquid Form and of Co-Administration of Enteral Nutrition

Serena Logrippo [1,2], Roberta Ganzetti [3], Matteo Sestili[4], Diego Romano Perinelli[5], Marco Cespi[5]* and Giulia Bonacucina[5].

[1] Hospital Pharmacy, Santa Maria della Stella Hospital, USL Umbria 2, 05018 Orvieto, Italy; serena.logrippo@sanita.marche.it
[2] Hospital Pharmacy, Engles Profili Hospital, AST Ancona, 60044 Fabriano, Italy;
[3] Hospital Pharmacy, Carlo Urbani Hospital, AST Ancona, 60035 Jesi, Italy; roberta.ganzetti@sanita.marche.it
[4] Territorial Pharmaceutical Service, AST Ancona, 60035 Jesi, Italy; matteo.sestili@sanita.marche.it
[4] CHIP Building, School of Pharmacy, University of Camerino, via Madonna delle Carceri, 62032 Camerino, Italy; diego.perinelli@unicam.it (D.R.P.); giulia.bonacucina@unicam.it (G.B.)

* Correspondence: marco.cespi@unicam.it; School of Pharmacy, University of Camerino, via Madonna delle Carceri, 62032 Camerino, Italy.

Table S1: Composition of the two nutrition formulae used in the study as provided by the manufacturers.

Values for 100 mL of product	Nutricomp® Standard Fiber (NSF)	Nutrison® 1Kcal/mol (NWF)
Energy (Kcal)	104	100
Protein (g)	3.8	4.0
Fat (g)	3.3	3.9
Saturated	0.9	1
Mono-unsaturated	1.1	2.2
Poly-unsaturated	1.3	0.7
EPA	0.04	0.02
DHA	0.03	0.014
Carbohydrate (g)	13.8	12.3
Sugar	1.5	0.7
Fiber (g)	1.5	/
